# On *Aethiopomyia* Malloch (Diptera, Muscidae) with the revision of the type specimens deposited in the Museum für Naturkunde, Berlin (Germany) with a key to species

**DOI:** 10.3897/zookeysw.926.49210

**Published:** 2020-04-13

**Authors:** Viviane Rodrigues de Sousa, André Fontinelle Magalhães Pereira, Márcia Souto Couri

**Affiliations:** 1 Museu Nacional, Quinta da Boa Vista, São Cristóvão, Rio de Janeiro, 20.940–040, RJ, Brazil Museu Nacional Rio de Janeiro Brazil; 2 Conselho Nacional de Desenvolvimento Científico e Tecnológico fellow, Brazil Conselho Nacional de Desenvolvimento Científico e Tecnológico Brasil Brazil

**Keywords:** Afrotropical, diversity, morphology, taxonomy

## Abstract

*Aethiopomyia* Malloch (Diptera, Muscidae) is a small genus with occurrence restricted to the Afrotropical region. Only five species are currently known in this genus: *A. patersoni* Zumpt, 1969, *A. williamsi* Snyder, 1951, *A. arguta* (Karsch, 1879), *A. steini* Curran, 1935, and *A. gigas* (Stein, 1906). All *Aethiopomyia* species are large, reaching up to 15 mm, as in *A. patersoni* and the most visible differences among them are based in the color pattern of the body. The species are mostly yellow, with a broad scutum and abdomen, males and females are dichoptic, the anepimeron is haired and they have stubby spines on the upper side of the palpi. Phylogenetically, *Aethiopomyia*, together with two other genera restricted to the Afrotropical region, *Alluaudinella* Giglio-Tos and *Ochromusca* Malloch, appear to form a monophyletic group, defined by the presence of the remarkably short stubby spines on the upper side of the palpi. Four species deposited in the Museum für Naturkunde, Berlin (Germany) were analyzed; three of them are types. Diagnosis for all species, colored illustrations, male dissections and illustrations and a key to separate them are presented herein.

## Introduction

*Aethiopomyia* Malloch (Diptera, Muscidae) is a small genus restricted to the Afrotropical region. It was proposed by Malloch (1921) with a differentiated diagnosis from the allied genus *Alluaudinella* Giglio-Tos. Both genera have a mostly yellow and broad scutum and abdomen, dichoptic males, anepimeron haired, and the presence of stubby spines on the upper side of the palpi which, according to Malloch (1921), readily separates the two genera from their nearest allies. In the differentiated diagnoses from *Alluaudinella*, Malloch (1921) mentioned the following characters of *Aethiopomyia*: propleuron hairy in center; proepisternum bare, prosternum hairy, metanotum with fine hairs on lateral elevation, vein R_4+5_ setulose at base below and above. *Spilogaster gigas* Stein was originally designated as the type-species.

In the phylogenetic analysis made by Couri and Carvalho (2003), these two genera, together with *Ochromusca* Malloch appear to form a monophyletic group, defined by the presence of remarkably short stubby spines on the upper side of the palpi. The larva of *Ochromusca* and *Alluaudinella* feed on dead snails, while the larval habits of *Aethiopomyia* are not known. According to Skidmore (1985) the final larval instar of *Aethiopomyia* closely resembles those of *Ochromusca*, *Alluaudinella*, *Synthesiomyia* Brauer & Bergenstamm, and *Muscina* Robineau-Desvoidy.

Five species are currently known in the genus. *Aethiopomyia patersoni* Zumpt, 1969 is restricted to Tanzania and *Aethiopomyia williamsi* Snyder, 1951 is recorded from Kenya, Malawi, and Tanzania. *Aethiopomyia arguta* (Karsch, 1879), *Aethiopomyia steini* Curran, 1935 and *Aethiopomyia gigas* (Stein, 1906) are more widespread in the Afrotropical region (Pont 1980). Zumpt (1969) published a key for the identification of the five species, mostly based on the color pattern of scutum and abdomen, together with taxonomic notes.

Diagnosis for all species, colored photographs, male dissections, illustrations, and a key to separate them are presented herein.

## Materials and methods

All examined material belongs to the Museum für Naturkunde, Berlin (Germany) and were examined during a scientific visit of MSC during the years 2018–2019. Four of the five species were analyzed, *A. williamsi* and types of *A. arguta*, *A. gigas*, and *A. patersoni*. For *A. steini* we used the characters in the original description.

Color photos were made using Auto-Montage. Complementary line drawings to the ones presented by Zumpt (1969) of the male terminalia of *A. arguta* were made, and male and female terminalia of *A. gigas* were dissected and illustrated.

The terminology follows that of Cumming and Wood (2017).

## Results

### Key to *Aethiopomyia* species

**Table:** 

1	Palpus yellow (Fig. 8), abdomen almost all black with some grey pruinescence along the margins of the tergites (Fig. 7) [Sternite 5 quadrangular with 2 strong setae at middle (Fig. 21); cercal plate and surstyli as in Figs 22 and 23; aedeagus as in Figs 24 and 25]	***A. gigas* (Stein)**
–	Palpus reddish brown to brown, abdomen more reddish brown with tergites variable	**2**
2	Calypters yellowish	**3**
–	Calypters yellowish with brown margin or fulvous brown	**4**
3	Arista yellow (Fig. 3), femora yellow (Fig. 1), mid tibia with 2 posterior setae, abdomen reddish brown, tergites III–V with a median brown vitta, tergite IV brown laterally and tergite V broadly brown [aedeagus as in Fig. 20]	***A. arguta* (Karsch)**
–	Arista mostly brown, femora reddish, mid tibia with 4 or 5 posterior setae, third and fourth abdominal segments wholly black	***A. steini* (Curran)**
4	Fronto-orbital plate silvery-white pruinose (Fig. 13), scutellum dark brown (Fig. 12) [abdominal sternites with strong setae]	***A. patersoni* Zumpt**
–	Fronto-orbital plate greyish brown (Fig. 17), scutellum light brown (Fig. 16) [abdominal basal sternites hairy, others with numerous long, ventrally directed setae in male]	***A. williamsi* (Snyder)**

### Aethiopomyia arguta

Taxon classificationAnimaliaDipteraMuscidae

(Karsch, 1879)

4EF17463-B4A9-525F-8B62-F0CCD8166137

Figures 1–5 , 20

#### Lectotype.

♂; **paralectotype** ♂ (see Pont and Werner 2006: 23–24 for details)

#### Diagnosis.

***Length of body*.** 11.0–12.0 mm (♂). ***Head*.** ♂ frons narrow, with the same width of frontal triangle. Frons and fronto-orbital plate dark brown. Parafacial, face and gena reddish yellow. Ocellar setae short. Gena very thin. Pedicel, postpedicel and arista yellow. Postpedicel ca. 4 × as long as wide. Arista long; plumose. Palpus brown, filiform. ***Thorax*.** Scutum reddish yellow-brown, with 1–3 incomplete brown white dusted vittae presuturally. Dorsocentrals 2+4. Katepisternals 1+2. Anepimeron setulose. Katatergite setulose. Lower calypter broad, ca. 3 × as long as the upper one. Haltere yellow. Calypters yellowish. ***Legs*.** Femora yellow; tibiae and tarsi brown. Fore tibia without median seta. Mid tibia with two posterior setae in middle third. ♂. Hind tibia with two anterodorsal and two or three anteroventral very fine setae. Pulvilli long and very enlarged (Fig. 4). ***Wing*.** Uniformly smoky yellowish. Costal spine not distinct. ***Abdomen*.** Robust, reddish brown; tergites III–V with a median brown vitta; tergite IV brown laterally and tergite V broadly brown. Rows of strong setae on margins of tergites IV and V and on disc of tergite V. Abdominal sternites with thin setae. Sternite 6 asymmetrical. ***Terminalia*.** Aedeagus as in Fig. 20.

#### Note.

The species was keyed by Zumpt (1969). Cercal plate and surstylus as in Zumpt (1969: fig. 3). We present complementary drawings of the terminalia, that are in the same slide prepared by Zumpt (1969).

**Figures 1–19.**
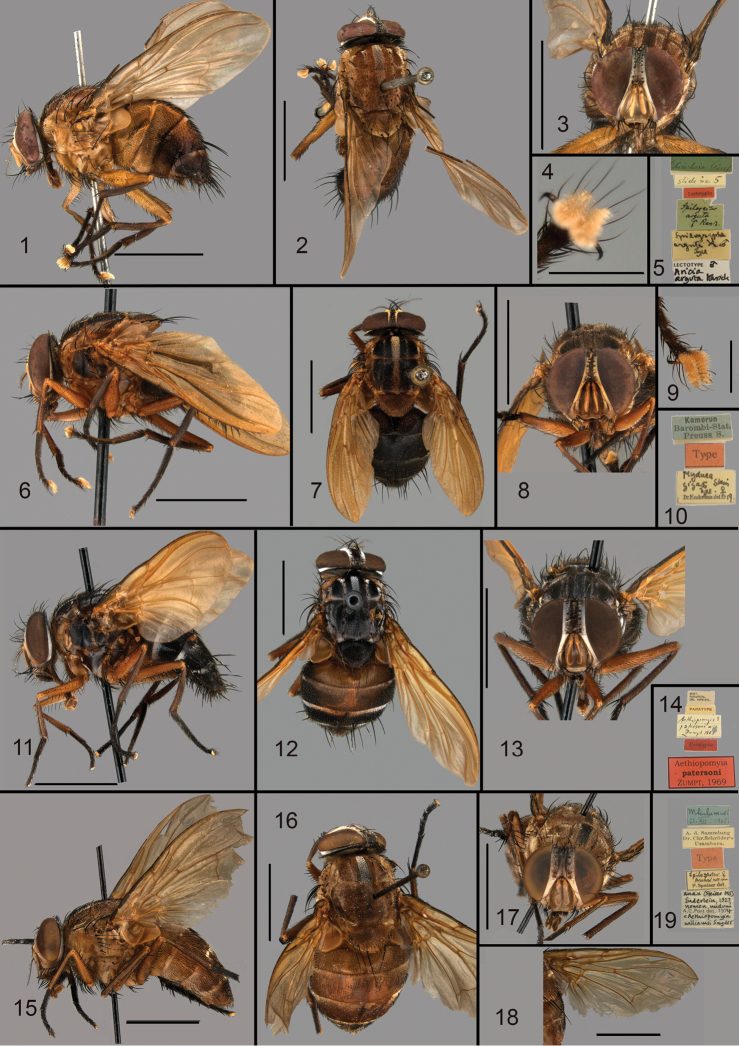
**1–5***Aethiopomyia arguta* (Karsch, 1879) lectotype ♂ of *Aricia arguta* Karsch: **1** lateral view **2** dorsal view **3** head frontal view **4** pulvilli **5** labels **6–10***Aethiopomyia gigas* (Stein, 1906) syntype ♂: **6** lateral view **7** dorsal view **8** head frontal view **9** pulvilli **10** labels **11–14***Aethiopomyia patersoni* Zumpt, 1969 Paratype ♀: **11** lateral view; **12** dorsal view **13** head frontal view **14** labels **15–19***Aethiopomyia williamsi* (Snyder, 1951) ♀ of *Lophomala anax* Enderlein, 1927, nomen nudum: **15** lateral view **16** dorsal view **17** head frontal view **18** wing **19** labels. Scale bars: 5 mm (**1–3, 5, 6–8, 10–19**); 1 mm (**4, 9**).

### Aethiopomyia gigas

Taxon classificationAnimaliaDipteraMuscidae

(Stein, 1906)

89B0D44C-B987-5DD2-A0EC-351CE98216FD

Figures 6–10 , 21–30

#### Syntypes.

1 ♂, 2 ♀ (see Pont and Werner 2006: 51 for details)

#### Diagnosis.

***Length of body*.** 11.0–15.0 mm (♂♀). Similar to *A. arguta*, differing as follows: ***Head*.** Frons, fronto-orbital plate, parafacial, face, and gena reddish yellow, silver pruinose under certain lights. Palpus yellow. ***Thorax*.** Scutum ground color yellow, four broad black vittae laterally and one median white-silver pruinose vitta. Scutum laterally yellow, scutellum yellow. Pleurae mostly yellow; anepisternum and anepimeron mostly dark brown, Lower calypter broad, ca. 2.5 × as long as the upper one. Haltere yellow. Calypters yellowish with brown margins. ***Legs*.** Color and chaetotaxy as in *A. arguta*. ***Abdomen*.** Almost all black, with some grey pruinescence along the margins of the tergites, under certain lights. Abdominal sternites with strong setae. Sternite 5 quadrangular with two strong setae at middle (Fig. 21). ***Terminalia*** ♂. Cercal plate and surstylus as in Figs 22 and 23; aedeagus as in Figs 24 and 25. ***Ovipositor*** as in Figs 26 and 27; only two spermathecae found (Fig. 28). ***Larva*.** One big larva found in ♀ abdomen (Fig. 29); detail of spiracle as in Fig. 30. Cuthbertson (1938 in Skidmore 1985) recorded that the ♀ deposits a single late instar larva.

#### Note.

The species was keyed by Zumpt (1969). One ♂ and one ♀ (not types) from Spanish Guinea were dissected and illustrated. ♂: Uellebg. Benitogbt. /15–31.i.07 / G. Tessmann S. G.; ♀: Alou Benitogbt/16–31.vii.06/ Tessmann S. G. Recently, O’Hara et al. (2013) transferred *Paleotachina* Townsend from Tachinidae to Muscidae and placed in synonymy with *Aethiopomyia* and *Paleotachina smithii* Townsend, type species of *Paleotachina*, was synonymized with *A. gigas*.

### Aethiopomyia patersoni

Taxon classificationAnimaliaDipteraMuscidae

Zumpt, 1969

FE1EDA2D-C4D3-54EF-AF51-9858F06586AF

Figures 11–14

#### Paratypes.

2 ♀ (see Pont and Werner 2006: 81 for details)

#### Diagnosis.

***Length of body*.** 11.0–14.0 mm (♀). Very similar to *A. gigas*, differing as follows: ***Head*.** Fronto-orbital plate silvery white pruinose. Palpus dark brown. ***Thorax*.** Scutum ground color brown, four broad black vittae laterally and one median white-silver pruinose vitta. Scutum laterally and scutellum dark brown. Pleurae dark brown, Lower calypter broad, ca. 3 × as long as the upper one. Haltere yellow. Calypters yellowish, the upper one with brown margin. ***Legs*.** Color and chaetotaxy as in *A. arguta*. ***Abdomen*.** Tergites I–III almost reddish brown; tergites IV and V almost all black. Abdominal sternites with strong setae.

#### Note.

The species was keyed Zumpt (1969). Cercal plate and surstylus as in Zumpt (1969: figs 1, 2). Only females seen.

### Aethiopomyia steini

Taxon classificationAnimaliaDipteraMuscidae

(Curran, 1935) (not seen)

624B5AAC-524E-5B9B-AFAC-6C1D20B9BB8F

#### Diagnosis

(characters from Curran (1935) original description). Length of body. 9.5–11.5 mm. ***Head*.** black, face and lower third of frons reddish with silver-white pruinescence. Frontal vittae brownish; palpus reddish brown; antenna yellow and arista mostly brown. ***Thorax*.** scutum reddish, darker above, with an incomplete median vitta and the lateral margins whitish pruinose, posteriorly with reddish brown pruinescence, the two broad, shiny, ferruginous vittae more or less divided by a thin yellowish pruinose line in front of the suture. Haltere reddish yellow. ***Legs*.** Reddish, posterior, and middle tibiae more or less brown and tarsi black. Mid tibia with four or five posterior setae. ***Abdomen*.** basal two abdominal segments rusty reddish, the third and fourth black.

Female. Differs in frontal vitta reddish brown above; no orbitals; scutum with very poorly defined dark vittae, almost unicolorous.

Types. Holotype ♂; 3 paratypes ♂ (all from Eden, Cameroon); Allotype ♀ (Sierra Leone) (Zumpt 1969). Not seen.

#### Note.

The species was keyed Zumpt (1969). According to Curran (1935)*A. steini* is very similar to *A. gigas*, differing by having the median vittae reddish and much finer setae on the sternite. Also differs from *A. arguta* by having the third and fourth abdominal segment wholly black.

### Aethiopomyia williamsi

Taxon classificationAnimaliaDipteraMuscidae

(Snyder, 1951)

317A8E28-0B94-57DC-A514-3EE2332A6AB5

Figures 15–19

#### Material examined.

2 ♀ of *Lophomala anax* Enderlein, 1927, nomen nudum (see Pont and Werner 2006: 20 for details)

#### Diagnosis.

***Length of body*.** 12.0–14.0 mm (♀) ***Head*.** Fronto-orbital plate greyish brown Palpus dark brown. ***Thorax*.** Scutum brownish yellow, with a median silvery grey vitta, more visible pre-suturally. Scutellum light brown, paler at tip. Calypters and halteres fulvous brown. ***Legs*.** Brownish yellow; tarsi dark brown. ***Abdomen*.** Brownish yellow with a dark dorsal median vitta on tergites IV and V. Abdominal basal sternites hairy, others with numerous long, ventrally directed setae in ♂ (from the original description; ♂ not seen).

#### Note.

The species was keyed by Zumpt (1969). Zumpt (1969: 166) doubted if this species is specifically different from *A. arguta*, as, according to him, they differ only by the thicker setae on abdominal sternites in *A. williamsi*. More specimens must be examined to elucidate the specific status of the species.

**Figures 20–30.**
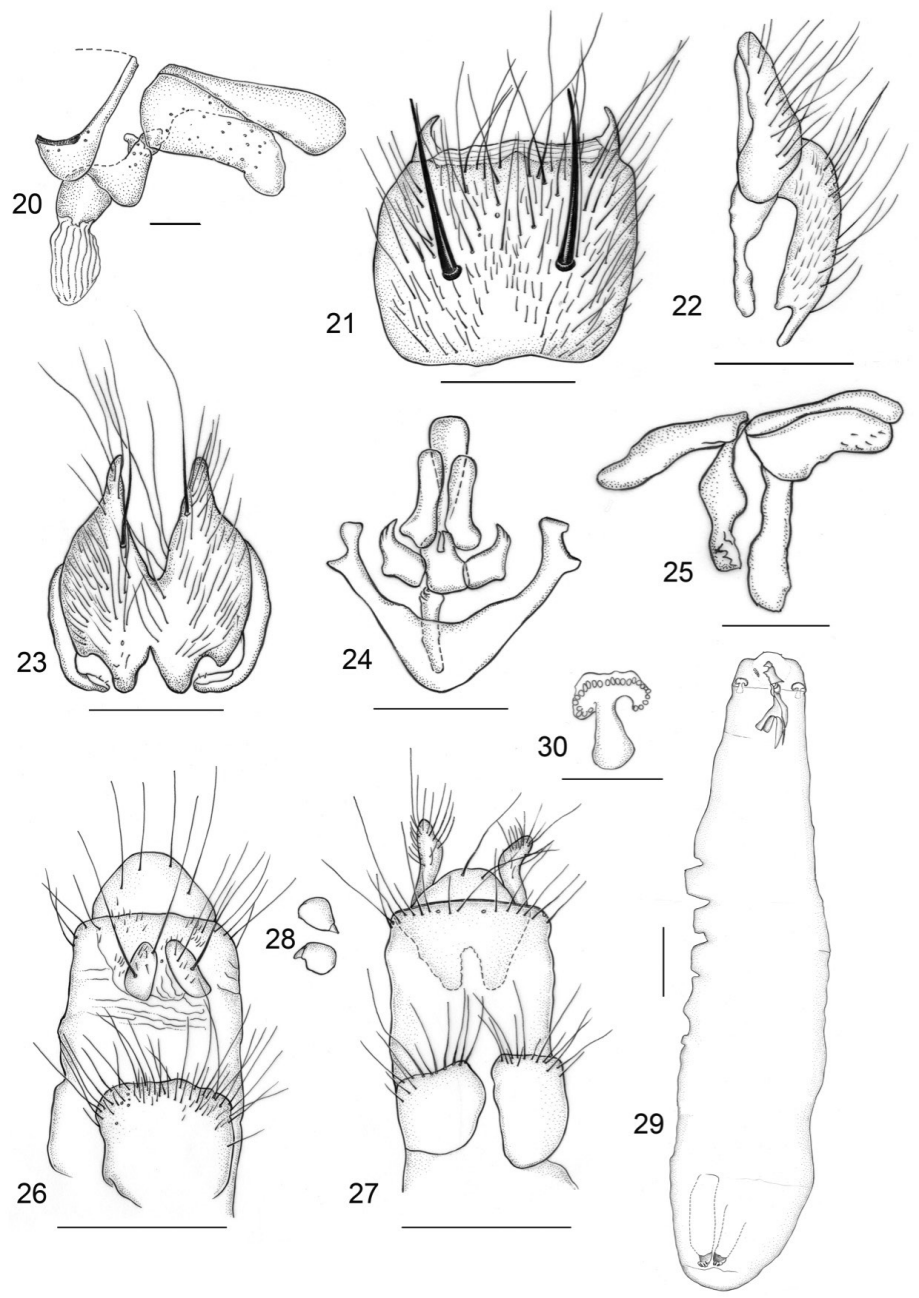
*Aethiopomyia arguta* (Karsch, 1879) **20** aedeagus lateral view. *Aethiopomyia gigas* (Stein, 1906) **21** Sternite 5 **22** cercal plate, lateral view **23** cercal plate, dorsal view **24** aedeagus dorsal view **25** aedeagus lateral view **26** ovipositor, dorsal view **27** ovipositor, ventral view **28** spermathecae **29** larva **30** detail of the anterior spiracle of larva. Scale bars: 0.2 mm **20–27, 29, 30**; 0.1 mm **20, 28**.

## Supplementary Material

XML Treatment for Aethiopomyia arguta

XML Treatment for Aethiopomyia gigas

XML Treatment for Aethiopomyia patersoni

XML Treatment for Aethiopomyia steini

XML Treatment for Aethiopomyia williamsi
